# Evaluation of Cytotoxic Effects of Various Endodontic Irrigation Solutions on the Survival of Stem Cell of Human Apical Papilla

**DOI:** 10.22037/iej.2016.7

**Published:** 2016

**Authors:** Narges Farhad Mollashahi, Eshaghali Saberi, Hamed Karkehabadi

**Affiliations:** a *Oral and Dental Diseases Research Center, Department of Endodontics, Dental School, Zahedan University of Medical Sciences, Zahedan, Iran; *; b * Department of Endodontics, Dental School, Hamedan University of Medical Sciences, Hamedan, Iran*

**Keywords:** Apical Papilla, Cytotoxicity, Irrigating Solution, MTT Assay, Stem Cells

## Abstract

**Introduction:**

Root canal disinfection is an important step in regenerative endodontic treatments. An ideal irrigating solution must have high antimicrobial activity and minimum cytotoxicity. This study sought to assess the effect of some irrigating solutions on stem cells from the human apical papilla (SCAP) after different periods of exposure.

**Methods and Materials:**

Stem cells were isolated from immature, impacted mandibular third molars, transferred to 24-well plates, randomly divided into 6 experimental groups and exposed to BioPure MTAD Cleanser, QMix, 17% EDTA, 2% chlorhexidine (CHX), 5.25% sodium hypochlorite (NaOCl), sterile saline and untreated control group. Cytotoxicity of these solutions was assessed after 1, 5 and 15 min of exposure using the methyl thiazol tetrazolium (MTT) assay. Data were statistically analyzed using repeated measures ANOVA. Level of significance was set at 0.05.

**Results:**

The mean percentage of viable cells in all experimental groups was significantly different from the control and sterile saline groups at all the time points (*P*<0.0001). The mean percentage of viable cells significantly decreased over time in NaOCl, QMix, EDTA and MTAD groups, but no significant reduction was noted in CHX group. At all the time points the highest and the lowest cytotoxicity were seen in MTAD and normal saline groups, respectively. Cytotoxicity of the understudy materials from the highest to the lowest was as follows: MTAD>EDTA>QMax=NaOCl>CHX> sterile saline.

**Conclusion:**

Chlorhexidine had the lowest cytotoxicity compared to EDTA, MTAD, QMix and NaOCl and its cytotoxicity did not change over time compared to other solutions.

## Introduction

Annually, millions of teeth undergo root canal treatment. Under ideal circumstances and highest standards, endodontic treatment has over 90% success rate in preservation of teeth. However, many teeth are non-restorable due to resorption, fracture, incomplete roots and severely damaged crowns due to caries and are replaced with prostheses such as dental implants [1, 2].

A new approach for tooth preservation has been proposed based on regenerative treatments using tissue engineering techniques and has been suggested as an alternative to preserve structurally compromised teeth. Tissue engineering is a multidisciplinary approach based on the principles of engineering and biology and aims to preserve and improve the function of tissues. The three key elements of tissue engineering include stem cells, morphogens and scaffold of the extracellular matrix [2, 3]. 

Regenerative endodontic treatment aims to replace the lost or damaged structures such as dentin, root structures and pulp-dentin complex cells [2, 3]. The simplest method for pulp tissue regeneration is pulp regeneration over the infected or necrotic tissue. Attempts to regenerate the pulp tissue under these conditions have all failed. Stem cells of the pulp, periodontal tissue and fibroblasts do not adhere or proliferate in infected root canal system [1]. Disinfection of the root canal system is an important step in regenerative endodontic treatment. According to the preclinical and case report studies, a successful regenerative treatment of a tooth with a necrotic pulp requires disinfection of the root canal spaces [4]. Several clinical approaches have been recently suggested for preservation and stimulation of dental stem cells, mostly focusing on chemical disinfection of the root canal system with different concentrations of sodium hypochlorite (NaOCl), a combination of NaOCl and chlorhexidine (CHX) and the triple antibiotic paste (TAP) [4-7]. These irrigants are used because of their known bactericidal and bacteriostatic effects. At the first study, Trevino *et al. *[5] highlighted that commonly used irrigants have a profound long lasting effect on survival of stem cells of human apical papilla (SCAP). They compared the effects of four irrigation protocols on the viability of SCAP cultured in platelet rich plasma (PRP) within the root canals *in vitro* and concluded that 17% EDTA followed by 17% EDTA and 6% NaOCl best supported and preserved the stem cells compared to 2% CHX. They showed that irrigants alone significantly affected the viability of *STRO-I* enriched stem cells.

An *in vitro* study subjected the human dental pulp stem cells to 0.04, 0.08, 0.16 and 0.33% NaOCl for 5, 10 and 15 min. The results showed that by decreasing the concentration of NaOCl, number of viable cells increased. Also, 0.04% NaOCl maintained the viability of cells at all time points [6]. 

The effect of 10 irrigating solutions and chelating agents on adhesion of stem cells to the root surfaces indicated that the number of adhered stem cells correlated with the cytotoxicity of the irrigant and it was stated that a biocompatible irrigant is required to improve the adhesion of stem cells [7]. 

Beside many commonly used irrigating solutions such as NaOCl and CHX, there many commercial multifunctional mixtures available for this purpose. QMix is a 2-in-1 solution containing a bisbiguanide antimicrobial agent (2% CHX) and a polyaminocarboxylic acid calcium-chelating agent (17% EDTA) [8]. Biopure MTAD (Dentsply, Tulsa Dental, Tulsa, OK, USA) is a mixture of a tetracycline isomer, citric acid, and a detergent (tween 80). These solutions have been successfully used in disinfection of root canal system [9-12].

Despite different therapeutic strategies in regenerative endodontic treatments, these treatments do not follow a specific standard; treatment protocols are implemented without adequate knowledge about the effects of disinfection methods on the viability of stem cells. These materials may be cytotoxic for stem cells and their cytotoxic effects on periodontal ligament stem cells (PDLSCs), cultured fibroblasts and stem cells of human exfoliated deciduous teeth (SHEDs) have been reported [5]. However, it is necessary to evaluate the effects of each of the chemical agents used in regenerative procedures on SCAP besides their better known antimicrobial properties. Thus, this study aimed to assess the effect of BioPure MTAD, QMix, 17% EDTA, 2% CHX, 5.25% NaOCl, on SCAP after different periods of exposure.

## Materials and Methods

The study protocol was approved in the ethics committee of Zahedan University of Medical Sciences (Grant No.: IR.ZAUMS.REC.1393.6251). Stem cells were isolated from two immature, impacted mandibular third molars of a healthy 19 year-old female patient. The patient was informed about the study objectives and signed a written informed consent. Immediately after extraction, the teeth were rinsed with phosphate buffered saline (PBS) solution (Gibco BRL, Grand Island, NY, USA) and stored in this sterile solution. Stem cells were then isolated from the apical papilla of the tooth by enzymatic digestion using type I collagenase (2mg/mL)(Worthlington Biomedical, Lakewood, NJ, USA) and placed in Dulbecco’s modified Eagle’s medium (DMEM). To obtain more cells, cells were re-cultured in culture medium containing 15% fetal bovine serum (FBS) (Gibco, Grand Island, NY, USA). This cell line was cultured in culture medium containing 10% DMEM/bovine serum in sterile cell culture flasks (SPL Life Science, Gyeonggi-do, South Korea). During the process of cell culture, the culture medium was refreshed every 2-3 days and cells were passaged after one week. After four passages, cells reached adequate confluence for cytotoxicity testing. Next, stem cells were transferred to 24-well plates and randomly divided into 6 experimental groups and subjected to BioPure MTAD Cleanser (Dentsply, Tulsa Dental, Tulsa, OK, USA), QMix^TM^ 2 in 1 (Dentsply, Tulsa Dental, Tulsa, OK, USA), 17% EDTA (MD-cleanser, Meta Biomed, Chungju, Korea), 2% CHX (Clorhexidina S, Dentscare LTDA, Joinville, SC, Brasil**)**, 5.25% NaOCl (Sehat, Tehran, Iran) and sterile saline. Stem cells cultured in DMEM were used as a control group. Cytotoxicity of the materials was assessed after 1, 5 and 15 min of exposure using the Mosmann’s Tetrazolium Toxicity (MTT) assay. 

The MTT solution was prepared by dissolving 5 mg of 3-(4,5-dimethyl-2-thiazolyl)-2, 5-diphenyl-2H-tetrazolium bromide (Sigma-Aldrich Co., St. Louis, MO, USA) in 1 mL of PBS. After filtering, this solution was diluted 1 to 10 using DMEM; 400 μL of the diluted MTT solution was added to each well and plates were incubated at 37^°^C under 5% CO_2_ and 95% humidity for 4 h. The supernatant in each well was gently extracted and replaced with 400 μL of dimethyl sulfoxide (DMSO, Gibco BRL, Grand Island, NY, USA). After dissolution of formazan crystals, optical density of the solution was read at 540 to 690 nm wavelength using an Elisa Reader (BioTek, Winooski, VT, USA). The intensity of color generated correlated with the percentage of viable (survived) cells. Data were analyzed using GraphPad Prism software (GraphPad Software, San Diego, CA, USA) at different time points *via* repeated measures ANOVA followed by Bonferroni test. Level of significance was set at 0.05.

## Results

The Bonferroni test was used to compare different groups. The results showed that the difference in the mean percentage of viable cells between the study groups and the control and sterile saline groups was statistically significant (*P*<0.0001). No significant difference was noted between the control and saline groups (*P*<0.05). At 5 min, a significant difference was noted in the mean number of viable cells between the CHX and MTAD groups (*P*<0.01). After 15 min, the mean number of viable cells in the CHX group was significantly different from that in the NaOCl (*P*<0.05), QMix (*P*<0.05), EDTA (*P*<0.05) and MTAD (*P*<0.0001) groups. 

**Figure 1:**
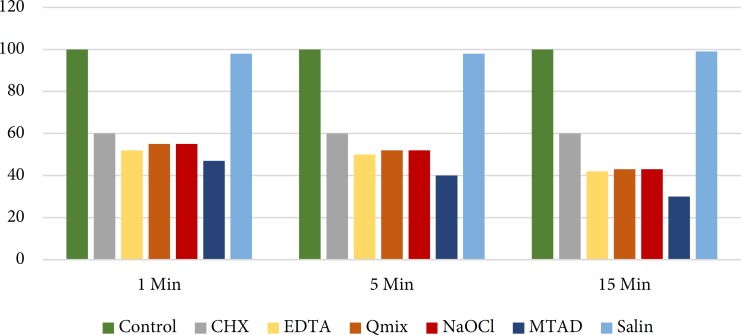
Comparison of the percentage of cell viability in different groups at 1, 5 and 15 min

Over time, no statistically significant change occurred in the mean number of viable cells in the CHX group but in NaOCl, QMix, EDTA and MTAD groups, the mean number of viable cells decreased over time. The difference in the percentage of viable cells between 1 and 15-min time points was only significant in NaOCl (*P*<0.0001), QMix (*P*<0.0001) and EDTA (*P*<0.05) groups (Figure 1). The highest cytotoxicity (highest rate of cell death) belonged to MTAD group at all the time points. In MTAD group, a significant difference was noted in the mean number of viable cells at 1 and 5 min (*P*<0.05) and 1 and 15 min (*P*<0.0001). The lowest cytotoxicity (lowest number of cell death) belonged to the sterile saline group. 

## Discussion

In the current study, cytotoxicity of NaOCl, EDTA, MTAD, CHX and QMix against SCAP was assessed using the MTT assay. A successful endodontic treatment depends on efficient mechanical and chemical debridement of the root canal system. Mechanical instrumentation of infected canals of immature roots is contraindicated due to the presence of fragile, under-developed dentinal walls. Thus, chemical debridement is the main technique of disinfection in regenerative endodontic treatments [13]. 

Unfortunately, there is no standard treatment protocol for regenerative treatments and different irrigating solutions are used for root canal disinfection. An ideal irrigant must have excellent antimicrobial property and minimum cytotoxicity [14, 15]. 

Several methods are used for assessment of cytotoxicity including the flow cytometry, the MTT or XTT, WST-1, WST-8 assay, and assessment of lactate dehydrogenase (LDH) activity, each having their own advantages and disadvantages. The MTT assay was introduced by Mosmann in 1980 [16, 17]. This method is used as a standard technique for assessment of the cytotoxicity of new biomaterials. This method evaluates the capability of viable cells in converting the water-soluble tetrazolium salts to the insoluble formazan crystals *via* the activity of mitochondrial dehydrogenase enzymes. This method assesses the cytotoxicity of dental materials based on the changes in the number of viable cells, cell metabolism and cell morphology. This method is simple and reproducible and does not require radioisotopes. In this method, cell damage is underestimated and only cell death, in the apoptotic phase, is detected when cellular metabolism significantly decreases [14, 15, 17, 18]. 

Our results showed that the mean number of viable cells in MTAD, QMix, NaOCl and EDTA groups experienced a reduction over time. This reduction, compared to the control and sterile saline groups, was significant. In the CHX group, the mean number of viable cells was significantly lower than the control and sterile saline groups; but over time, no significant change occurred in the mean number of viable cells. Cytotoxicity of NaOCl, QMix, EDTA and MTAD increased over time. In other words, cytotoxicity in these groups was time-dependent. Comparison of the mean number of viable cells in the study groups showed that at all time points the highest cytotoxicity was seen in MTAD group while the lowest cytotoxicity was noted in the sterile saline group. The cytotoxicity of the materials from the highest to the lowest was as follows: MTAD>EDTA>QMix and NaOCl>CHX>sterile saline. Assessment of the cytotoxicity of materials *in vitro, *is completely cellular. Cultured cells compared to the periapical tissues are highly susceptible to the toxic effects of drugs [17]. Under *in vivo *conditions, materials are diluted with body fluids and their concentration decreases [19]. Also, they are eliminated by the function of phagocytes, vascular and lymphatic systems. On the other hand, the inhibitory effect of dentin on irrigants must be taken into account as well [5, 20]. Thus, in equal concentrations, the cytotoxicity of materials decreases over time in the clinical setting compared to *in vitro* [17, 21]. Therefore, our results may not be directly generalizable to the *in vivo* settings. 

The highest cytotoxicity in our study belonged to MTAD followed by EDTA. Yasuda *et al.* [14] showed that the cytotoxicity of MTAD against MC3T3-E1 osteoblast-like cells and periodontal ligament cells was lower than that of 5.25% NaOCl, 17% EDTA and 0.12% CHX. Cytotoxicity of these materials was evaluated on L929 fibroblasts during a 24-h period. In a study by Zhang *et al.* [15] MTAD showed lower cytotoxicity than 5.25% NaOCl and EDTA and higher cytotoxicity than 2.63%, 1.31% and 0.66% NaOCl. Ring *et al.* [7] demonstrated that the cytotoxicity of NaOCl/MTAD was slightly lower than that of NaOCl and NaOCl/EDTA. This indicated higher biocompatibility of MTAD than NaOCl. Another study also confirmed the cytotoxicity of 17% EDTA even when it was diluted to 0.1% [20]. The difference between the results was due to the difference in sensitivity of cell lines used or test conditions such as the use of different concentrations of materials and different assessment time points.

Trevino *et al.* [5] used an organotype model of root canal system to assess the effect of irrigants on the viability of SCAPs; their results were in contrast to our findings. They reported that 17% EDTA provided the best support for SCAP while protocols containing 2% CHX were devoid of viable stem cells. Such controversy between the results of the two studies may be due to the fact that an organotype model (compared to culture dishes) evaluates the interaction effects of host tissues (dentin and cementum) on stem cells. Dentin has physiological concentrations of growth factors affecting the proliferation and differentiation of undifferentiated mesenchymal cells. On the other hand, dentin is a unique combination of extracellular matrix proteins and surface molecules and these characteristics do not exist in culture media *in vitro*. 

EDTA stimulates the release of these growth factors from dentin and increases their bioavailability. Also, EDTA eliminates the smear layer and disinfects dentin and consequently, enhances the adhesion of stem cells [13]. Irrespective of the effect of EDTA on the release of bioactive molecules, apical extrusion of EDTA not only causes decalcification of periapical bone, but also may compromise neuroimmune regulations, even in very low concentrations [22]. Also, leakage of EDTA into the periapical tissues may inhibit the function of macrophages and decrease the periapical inflammatory reactions [23]. 

Contrary to EDTA, NaOCl inhibited the differentiation of SHEDs and dental pulp stem cells to pre-odontoblast cells *in vitro* and *in vivo*. NaOCl denatures the dentin-derived growth factors and inhibits their effect on differentiation and proliferation of mesenchymal stem cells [13]. 

QMix was one of the solutions evaluated in our study. This solution is a mixture of polyamino carboxylic acid, saline, bisbiguanide antimicrobial agent, calcium chelating agent and surfactant. It has antimicrobial properties and substantivity but cannot dissolve tissues. In our study, the cytotoxic effect of QMix was found to be highly similar to that of 5.25% NaOCl and higher than that of 2% CHX. In a study by Chandrasekhar *et al. *[24], QMix showed less cytotoxicity than 3% NaOCl, 2% CHX and 17% EDTA in rat subcutaneous tissues. However, their methodology and concentration of solutions used were different from our study.

The lowest cytotoxicity belonged to CHX after sterile saline. Cytotoxicity of CHX, in contrast to that of MTAD, EDTA, QMix and NaOCl, did not change significantly over time. In a previous study, 2% CHX showed residual antibacterial activity and was stronger than 5.25% NaOCl in this regard. Also, it showed lower cytotoxicity compared to 5.25% NaOCl [25]. In a study by Bajrami *et al.* [17], the cytotoxicity of 2% CHX was higher than that of MTAD and NaOCl against periodontal ligament fibroblasts of rats. The results of an *in vitro* study on the cytotoxicity of CHX against human gingival cells showed that the toxic potency of CHX depended on the composition of the exposure media, exposure dose and length of exposure [26]. Chlorhexidine does not seem to have long-term toxic effects on host tissues, but may cause an inflammatory response in these tissues.

Cytotoxicity assessment of materials with different methods yields different results. Thus, it is recommended to use different methods to increase the accuracy and reliability of results. The results of our *in vitro* study only showed toxicity at the cellular level. Assessment of different concentrations and compositions of irrigants *via* organotype root canal models and *in vivo* animal and clinical studies is necessarily required to evaluate their histocompatibility.

## Conclusion

Chlorhexidine had the lowest cytotoxicity compared to EDTA, MTAD, QMix and NaOCl and its cytotoxicity did not change over time compared to other solutions. These results can provide a key for choosing the irrigating solution in cases of pulp regeneration.
